# Controllable synthesis of graphene scrolls and their performance for supercapacitors†

**DOI:** 10.1039/c8ra02231c

**Published:** 2018-05-24

**Authors:** Lianlian Gao, Zhigang Zhang, Jinping Zhao, Jin Zhou, Zhichao Miao, Weijiang Si, Shuping Zhuo

**Affiliations:** School of Chemistry and Chemical Engineering, Shandong University of Technology Zibo 255049 P. R. China jpzhao@sdut.edu.cn siweijiang@sdut.edu.cn

## Abstract

A graphene scroll (GSC) is a new type of graphene-derivative material, that has widely attracted attention. However, the controllable preparation of GSCs is a major factor influencing their development and application. In this work, sodium citrate (SC) was added to a graphene oxide (GO) aqueous suspension and GSCs were controllably prepared on a large-scale by a cold quenching method. The results show that the number of scroll layers and the curling degree of the GSCs could be controlled by the quantity of SC added. The diameter of the GSCs increased when SC was added. Compared to the GSC without SC (265 nm), the average diameter of GSC(SC-40) (obtained by adding 40 mg SC to 100 mL GO solution (1 mg mL^−1^)) is 491 nm. When excessive SC was added, such as 100 mg, the average diameter reached 679 nm. Moreover, these GSCs were used as a supercapacitor electrode material and the electrochemical performance was tested. The specific capacitance of GSC(SC-40) (178 F g^−1^) is higher than that of the GSC without SC (107 F g^−1^) at the same current density of 1.0 A g^−1^. However, when a larger quantity of SC was added, the specific capacitance of the GSCs decreased. So, the number of scroll layers and the curling degree of the GSCs have a significant effect on the electrochemical properties of the supercapacitor.

## Abstract 2

In this work, sodium citrate (SC) was added to a graphene oxide (GO) aqueous suspension and GSCs were controllably prepared on a large-scale by a cold quenching method.

## Graphical abstract

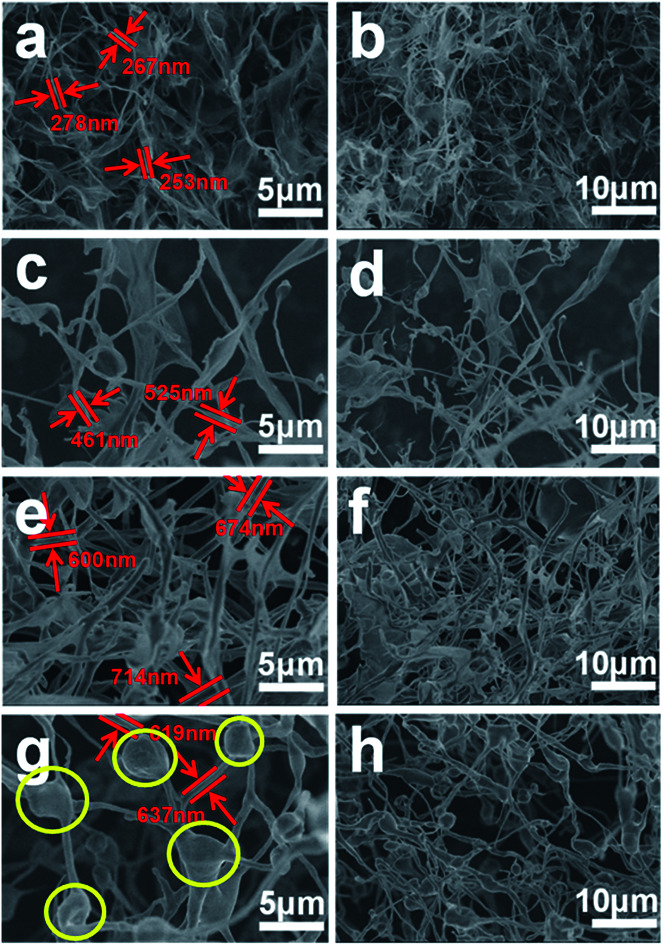

## 1. Introduction

A graphene scroll (GSC) is a one-dimensional (1D) nanostructure similar to a multiwalled carbon nanotube (MWCNT), which is a curled two-dimensional (2D) graphene sheet (GS).^1–4^ GSCs as a new type of graphene-derivative material have widely attracted attention due to their unique properties. Firstly, GSCs possess excellent electric properties due to the unclosed topological structure.^5,6^ In addition, the diameter of GSCs can be adjusted and some materials can be easily inserted into the interlayers of GSCs.^1^ These properties of GSCs mean they have great potential in energy storage such as in supercapacitors and lithium-ion batteries.^7–9^ For example, Fan *et al.* prepared GSCs through shock cooling by liquid nitrogen. Compared with GSs (108 F g^−1^), the specific capacity for the GSCs was 156 F g^−1^ at a current density of 1.0 A g^−1^.^10^ Also, Zeng *et al.* used a microexplosion method to synthesise GSCs that demonstrated a remarkable capacity of 162.2 F g^−1^ at a current density of 1.0 A g^−1^ in 6 M KOH aqueous solution, compared to a specific capacity of 110 F g^−1^ for GSs.^11^ Zhao *et al.* prepared a composite material of graphene nanoscroll (GNS) wrapped Fe_3_O_4_ nanoparticles (Fe_3_O_4_@GNSs) by cold quenching in liquid nitrogen. Fe_3_O_4_@GNSs was used as an electrode material for lithium-ion batteries and demonstrated a high specific capacity of 1010 mA h g^−1^.^12^ Li *et al.* synthesised TiO_2_(B)-G scrolls of GNS encapsulated TiO_2_(B) nanowires for lithium-ion batteries. The results showed that the specific capacity remained at 153 mA h g^−1^ after 300 cycles at 10C with a capacity retention of 94%.^13^

If GSCs are used as an electrode material, we must obtain GSCs with a controlled structure on a large-scale. At present, there are various preparation methods for GSCs, such as exfoliation-sonication of intercalated graphite,^14,15^ chemical vapor deposition (CVD),^16,17^ arc-discharge,^18^ high-energy ball milling of graphite,^19^*etc.* In these preparation methods, many impurities and defects are produced. The CVD method is expensive and it is difficult to achieve mass production. The arc-discharge and high-energy ball milling of graphite methods cannot guarantee the purity of the GSCs. These methods do not resolve the issue of large-scale preparation of GSCs. Therefore, the preparation method is a major factor restricting the development and application of GSCs. In our previous work, we found that the liquid-nitrogen quenching method is a very good method for the large-scale preparation of GSCs.^20^ But, in the previous work we could not control the number of scroll layers or the curling degree of the GSCs. So how can the preparation of GSCs be controlled?

As is known, there is a crucial relationship between the surface energy of the graphene oxide (GO) sheets and the process of rolling the GO sheets into GSCs.^4,21^ A surfactant is a kind of reagent that is able to change the surface properties of a material. Therefore, in our work, our first thought was whether the curling structure of the GSCs could be changed by adding a surfactant to the GO aqueous suspension. In our previous work, we found that sodium citrate (SC) has abundant functional groups and a short carbon chain, and can be used as a surfactant to assist the synthesis of a GSC composite.^22^ There are two advantages: firstly, SC has abundant functional groups and can change the surface properties of the GSs, which makes it easy to roll the GSs into GSCs; secondly, SC also has a short carbon chain which leads to a shorter diffusion path for ion transportation in the material, which improves the electrochemical performance.^22^ Therefore, during the experiment, we want to use SC as the surfactant to decorate the GSs in the aqueous suspension and control the curling structure of the GSCs by changing the quantity of SC added.

In this work, GSCs are prepared by a liquid-nitrogen quenching method and SC is added into the GO aqueous suspension during the preparation process to control the number of scroll layers and the curling degree of the GSCs. The scroll structure of the GSCs is controlled by changing the quantity of SC added. The morphology of the GSCs is characterized to explore the relationship between SC and the change in the scroll structure. Moreover, the electrochemical performance of the different GSC structures was tested. The results showed that the scroll structure and the electrochemical properties of the GSCs were closely correlated to the amount of SC added.

## 2. Experimental section

### 2.1. Materials

The graphite used was natural flake graphite (99%, 32 mesh, mean particle size of about 560 μm) which was purchased from Qingdao Tianheda Graphite Ltd. Co (Qingdao, China). All reagents were analytical reagents (AR) and were not further treated before use. Distilled water was used in this work.

### 2.2. Preparation of GSC and GSC(SC)

In this experiment, the natural flake graphite of 32 mesh was used as a raw material to prepare the large-area GO sheets in the GO aqueous suspension. The GO aqueous suspension was synthesized by a modified Hummers method according to a previously reported method.^23^ The suitable concentration of the GO aqueous suspension was about 1 mg mL^−1^. GSC materials were synthesized by a liquid-nitrogen quenching method and the main experiment content was as follows.

First, a certain quantity of SC was dispersed in the GO aqueous suspension (100 mL, 1 mg mL^−1^) and the mixed suspension was further dispersed by appropriate magnetic stirring and sonication. Then, the above uniform suspension was moved into tubes. The suspension was heated to 80 °C, and then was quickly put into liquid nitrogen. After the suspension had completely cooled, the tube was removed. Finally, water was evaporated from the cooled suspensions by vacuum freeze-drying to obtain graphene oxide scrolls with SC (GOSC(SC)), and the dry GOSC(SC) materials were reduced under a nitrogen atmosphere at a temperature of 600 °C to obtain GSC(SC). According to the different quantities of SC, the samples were named GSC(SC-40), GSC(SC-100), and GSC(SC-600), when 40 mg, 100 mg and 600 mg of SC was added into the GO aqueous suspension (100 mL, 1 mg mL^−1^), respectively.

A sample without SC was also prepared under the same experimental conditions, which was named GSC.

### 2.3. Material characterization

To analyze the morphology and structure of all the samples, scanning electron microscopy (SEM, Sirion 200FEI, Netherlands) and transmission electron microscopy (TEM, Tecnai F20, 200 kV) were carried out. The electrical conductivities were measured by the semiconductor resistivity of the powder tester (ST-2722). In addition, the structures were also observed by Raman spectroscopy (Labram HR-800, Horiba Jobin Yvon) and the surface chemical properties were characterized by X-ray photoelectron spectroscopy (XPS, VG ESCA2000). Nitrogen sorption of the prepared materials was tested using ASAP 2020 equipment (Micromeritics USA) to measure the specific surface area and the pore size distribution (PSD). The Brunauer–Emmett–Teller (BET) surface area (*S*_BET_) was calculated using the N_2_ adsorption isotherm data within a relative pressure of 0.05–0.25. The PSD was determined by applying the nonlocal density functional theory (NLDFT) model to the adsorption isotherms and assuming a slit-shaped pore.

### 2.4. Electrochemical measurements

In this report, all of the prepared samples were used as a supercapacitor electrode material in a three-electrode system to test the electrochemical properties. This three-electrode system contained a reference electrode (SE, a saturated calomel electrode (SCE)), a counter electrode (CE, a platinum foil electrode) and a working electrode (WE). The electrolyte was 6 M KOH aqueous solution. This test was carried out on a CHI660D electrochemical testing station (Chenhua Instruments Co. Ltd, Shanghai) at room temperature.

The working electrode was prepared as follows. Briefly, the electroactive material (90 wt%) and poly(vinylidene difluoride) (10 wt%) binder were mixed. Then, the resulting mixture was pressed onto a nickel foam collector at about 10 MPa, which was followed by drying at 60 °C for 16 h in a vacuum oven. Each electrode contained about 2.0 mg of the electroactive material and had a geometric surface area of about 1 cm^2^.

The electrochemical measurements of each as-prepared electrode included cyclic voltammetry (CV), galvanostatic charge–discharge and electrical impedance spectroscopy (EIS). CV tests were measured at different scan rates within a voltage range of 0 to −1.0 V (*vs.* SCE). Galvanostatic charge–discharge was tested at different current densities from 0.1 to 5.0 A g^−1^. EIS measurement was performed in the frequency range 0.01 Hz to 10 kHz on open circuit potential.

The corresponding specific capacitance was calculated from:1
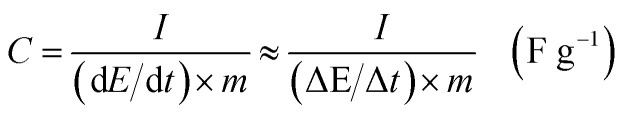
where *C* (F g^−1^) is the specific capacitance, *I* (A) is the constant discharging current, d*E*/d*t* indicates the slope of the discharging curves, and *m* (g) is the mass of the corresponding electrode material.

The corresponding specific capacitance was also calculated by the following formula:2
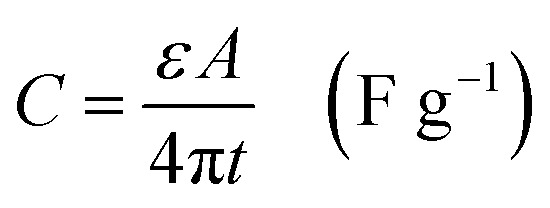
where *C* (F g^−1^) is the specific capacitance, *ε* is the equivalent permittivity, *A* is the surface area of the electrode material and *t* is the thickness of the double-layer.

## 3. Results and discussion

### 3.1. Structural characterization

**Fig. 1:**
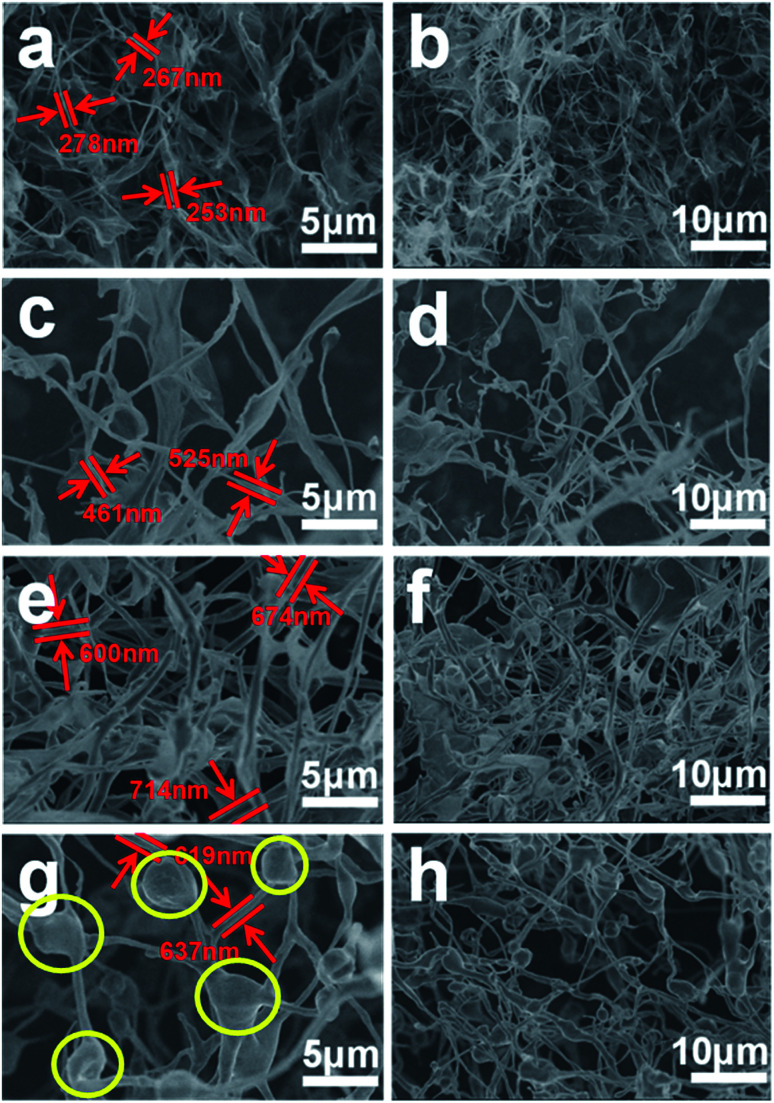
SEM images of GSC (a and b), GSC(SC-40) (c and d), GSC(SC-100) (e and f) and GSC(SC-600) (g and h).

**Fig. 2:**
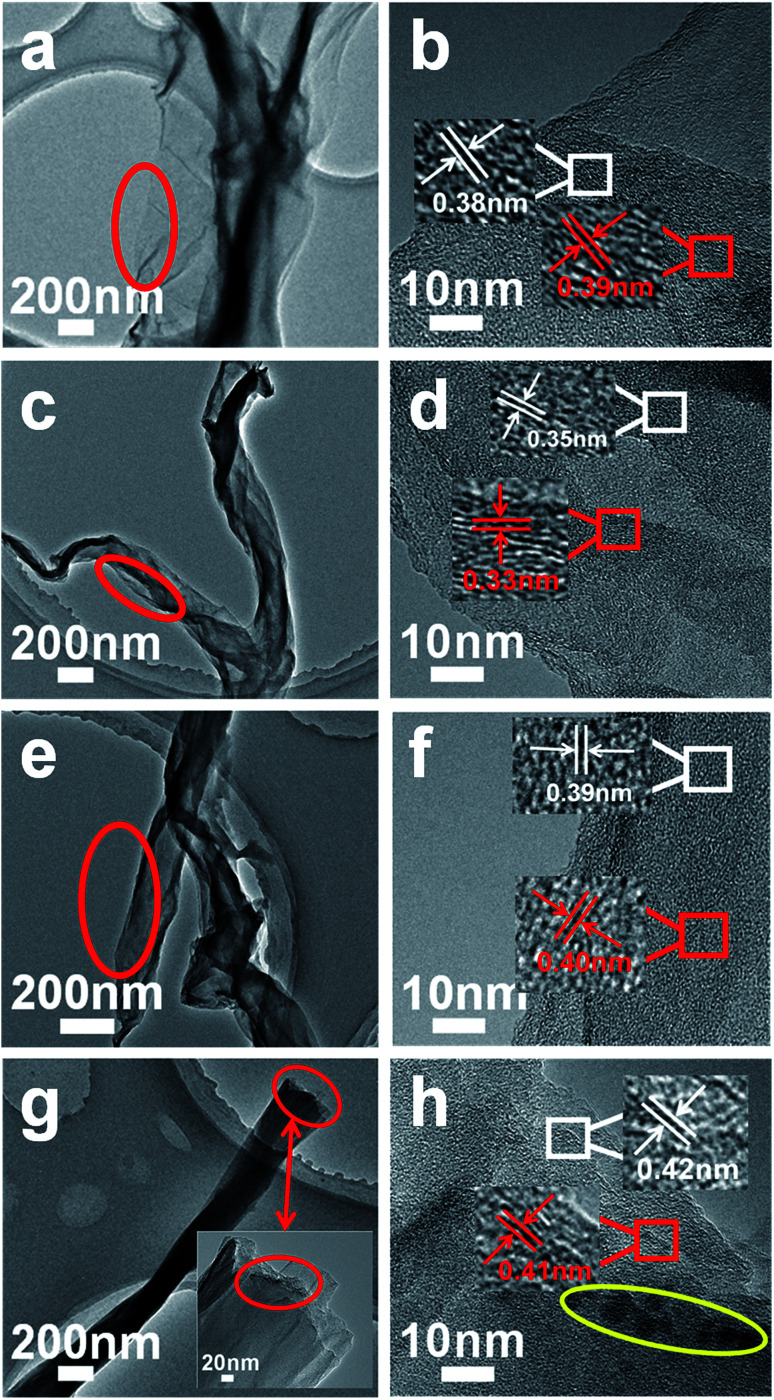
TEM images of GSC (a and b), GSC(SC-40) (c and d), GSC(SC-100) (e and f) and GSC(SC-600) (g and h).

The morphology and structure of the GSC, GSC(SC-40), GSC(SC-100) and GSC(SC-600) samples were tested by SEM and the results are shown in Fig. 1. We can clearly see that all of the samples have the common character of an unclosed topological structure which is a unique property of GSCs.^5,6^ Moreover, for all of the samples the GSCs cross each other and form a 3D network, which has been characterized in our previous work.^20^ More interestingly, we also noticed that with an increase in the amount of SC, the curled structure of the GSCs is obviously changed. In Fig. 1(a) and (b), without SC, the GSs are rolled up incompletely. For the GSC(SC-40) sample as shown in Fig. 1(c) and (d), most of the GSs are rolled up and the curling degree of the GSs is greater. When 100 mg and 600 mg SC are added, the GSCs are more curled. Moreover, for the GSC(SC-600) sample, SC decomposes incompletely and leads to many agglomerations (as shown in the yellow circle of Fig. 2(h)), which can also be seen in the TEM images shown in Fig. 2(g) and (h). Ten GSCs were chosen to measure the average diameter and the data are described in Fig. S1 and Table S1.† The results show that compared with GSCs (265 nm) without SC, the average diameter of GSC(SC-40) is 491 nm. However, for GSC(SC-100) and GSC(SC-600), the average diameter reaches 679 nm and 619 nm respectively. These results demonstrate that the curled structure of GSCs can be effectively controlled by changing the addition amount of SC. It is known that the formation of GSCs has a crucial relationship with the surface properties of the GSs during the process of rolling the GSs into GSCs. So the reason for these results is that the surfactant SC, that has abundant functional groups, can change the surface morphology of the GSCs.^22^

In order to verify the SEM analysis results, Fig. 2 shows TEM images of GSC (a and b), GSC(SC-40) (c and d), GSC(SC-100) (e and f) and GSC(SC-600) (g and h). First, it is clear in Fig. 2 that with an increase in the quantity of SC, the number of curled layers increases gradually (GSC(SC-600) has the most curled layers). Furthermore, it is worth noticing that the interlayer spacing is about 0.34 nm in the GSC(SC-40) sample, which is smaller than GSC (0.39 nm) due to the effect of SC.^22^ However, for the GSC(SC-100) and GSC(SC-600) samples, the interlayer spacing is 0.40 nm and 0.42 nm respectively, which is bigger than GSC, as shown in Fig. 2(e–h). The reason for this is that a moderate quantity of SC can enhance the interface between the GSs, which makes the interlayer spacing decrease, but a larger quantity of SC is not completely reduced and remains in the GSCs (as shown in the yellow circle of Fig. 2(h)), which makes the interlayer spacing increase. These results are consistent with the SEM analysis and this change may have a great influence on the electrochemical performance.

**Fig. 3:**
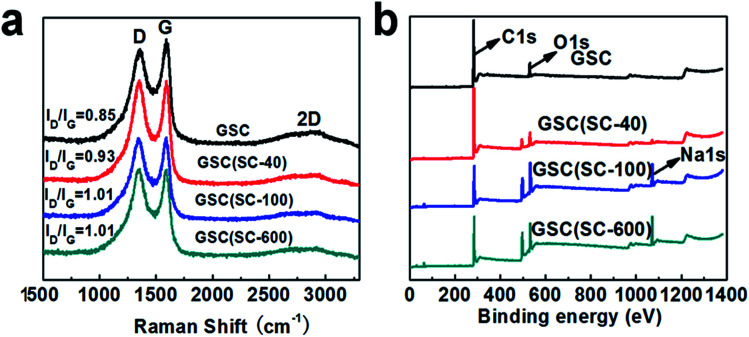
(a) Raman spectra and (b) XPS spectra for GSC, GSC(SC-40), GSC(SC-100) and GSC(SC-600).

Raman spectroscopy is a good tool to analyze the structure of carbon materials. Generally, there are three broad peaks in the Raman spectra, which are the D, G and 2D bands at about 1340, 1580 and 2800 cm^−1^ respectively.^24^ The 2D peak of graphitic materials is known to be extremely sensitive to the number of layers, and its shape is indicative of the number of layers per flake.^27,28^ After the addition of SC, the curled structure of the GSCs is changed, so the 2D peak becomes unremarkable. When the number of defects in carbon materials increases, the intensity of the D peak will increase and the intensity of G peak will decrease, which will result in an increase in the intensity ratio of the D and G bands (*I*_D_/*I*_G_).^25^ Therefore, we can use the ratio *I*_D_/*I*_G_ to measure the number of defects and the degree of disorder for the GSCs. Fig. 3(a) shows the representative Raman spectra of GSC, GSC(SC-40), GSC(SC-100) and GSC(SC-600) after thermal reduction. First of all, we can clearly see that there are two strong characteristic peaks for the D and G bands at around 1350 and 1580 cm^−1^ for all of the samples. In addition, after calculation, the *I*_D_/*I*_G_ values for the GSCs(SC) are bigger than that of GSC, which means that the addition of SC leads to more defects in the GSCs and the structure of the GSCs becomes more disordered.

**Fig. 4:**
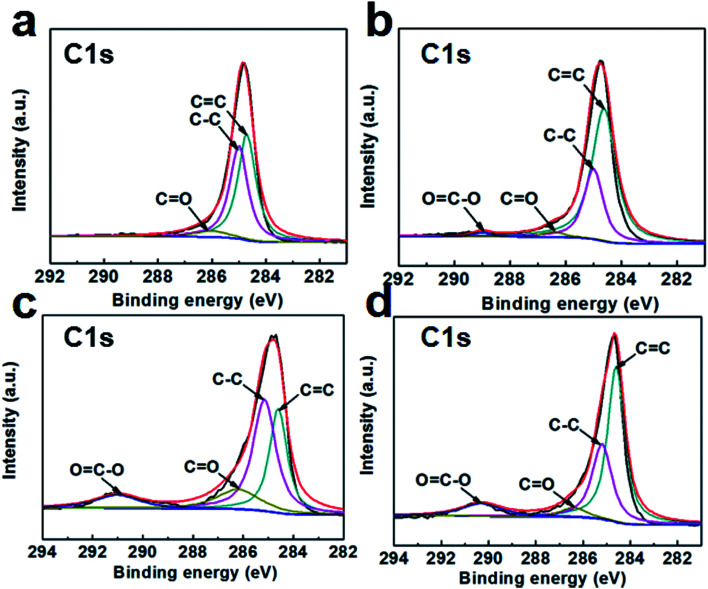
C1s XPS spectra of GSC (a), GSC(SC-40) (b), GSC(SC-100) (c) and GSC(SC-600) (d).

The XPS spectra are shown in Fig. 3(b) to analyze the elemental compositions of the GSC, GSC(SC-40), GSC(SC-100) and GSC(SC-600) samples. The results show that there are two obvious peaks for C and O for all of the samples. The content of C, O, and Na elements is listed in Table S2.† For GSC(SC-40), the C/O value is reduced to 9.67. However, in the GSC(SC-100) and GSC(SC-600) samples, the C/O value declines sharply to 3.18 and 2.82, respectively, and the atomic ratio of Na rises to 8.57 and 9.36 respectively. This indicates that when an excessive amount of SC is added, a large amount of the SC cannot be completely converted to carbon and still remains after thermal reduction, because the SC is tightly wrapped by the GSCs and is not easy to break down. This result is consistent with the above results. In addition, Fig. 4 shows the C1s spectra of the GSCs. The hetero-carbon components change dramatically. In Fig. 4(a), there are three different groups of C–C, C=C and C=O. When SC was added, the O=C–O group appears, owing to the function of SC as shown in Fig. S2.† Moreover, with an increase in SC, the O=C–O peak becomes stronger, which suggests that the residual amount of SC increases.

To further determine whether this structural change in GSCs may have a great influence on the electrochemical performance, N_2_ sorption isotherms and a pore size distribution plot of GSC(SC-40) are shown in Fig. S3.† It can be seen that all the prepared materials exhibit a typical type IV isotherm indicating that they have large mesopores or macropores. From the pore size distribution plot of GSC(SC-40) (Fig. S3(b)†), there are similarly large mesopores or macropores. As shown in Table. S3,† the specific surface area (*S*_BET_) increases from 207 m^2^ g^−1^ (GSC) to 318 m^2^ g^−1^ (GSC(SC-40)). When a large amount of SC was added, *S*_BET_ got very small. These structural changes may lead to a different electrochemical performance.

### 3.2. Electrochemical properties of the supercapacitor

**Fig. 5:**
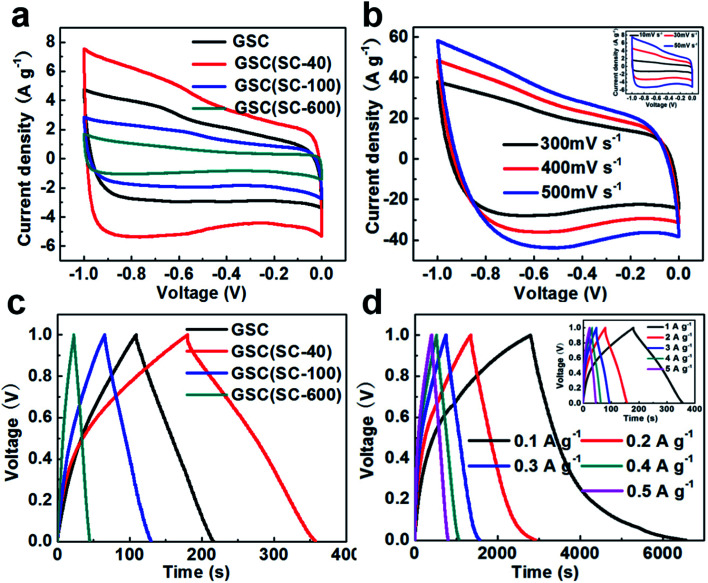
Electrochemical behavior in 6 M KOH electrolyte: CV curves of all the samples at a scan rate of 50 mV s^−1^ (a) and GSC(SC-40) at different scan rates (b); galvanostatic charge–discharge curves of all the samples at a current density of 1 A g^−1^ (c) and GSC(SC-40) at different current densities (d).

In order to explore the effect of SC on the electrochemical properties, all of the samples were used as supercapacitor electrode materials in 6 M KOH electrolyte employing a three-electrode system with a voltage window of −1.0 to 0 V, and the results are shown in Fig. 5. Fig. 5(a) shows CV curves of all the samples at the same scan rate of 50 mV s^−1^. It can be seen that all the CV curves are close to a rectangular shape, which indicates that the electrochemical behavior is more like an ideal EDLC. Compared with GSC, the GSC(SC-40) electrode exhibits a larger CV area, showing a higher specific capacitance. However, for the GSC(SC-100) and GSC(SC-600) samples, the rectangular area is smaller than for GSC, which means that the electrochemical performance could be restrained with an excessive amount of SC. These results suggest that GSCs with an appropriate amount of SC could show a better electrochemical performance. CV curves of the GSC(SC-40) sample at different scan rates of 10–500 mV s^−1^ are shown in Fig. 5(b). The GSC(SC-40) sample still exhibits an almost ideally rectangular shape even at a very high scan rate of 500 mV s^−1^, demonstrating that GSC(SC-40) has very good power capability. To compare the specific capacitance, Fig. 5(c) shows galvanostatic charge–discharge curves of all the samples within a potential window of 0 to −1.0 V at the same current density of 1 A g^−1^. It can be observed that all the triangular shapes are closely linear and symmetrical, displaying a good capacitive property. In addition, it is worth noting that the larger current density response and longer discharge time of GSC(SC-40) means it has higher specific capacitance than the other samples. According to formula (1) in the experiment section, the specific capacitance of GSC(SC-40) is 178 F g^−1^, which is much bigger than GSC (107 F g^−1^). But, the specific capacitance of GSC(SC-100) and GSC(SC-600) is only 64 and 22 F g^−1^, respectively. When 40 mg of SC was added, due to SC enhancing the interface between the GSs, the interlayer spacing (0.34 nm) became smaller and the formation of more electrical double layers caused an increase in the specific capacitance.^22^ In formula (2) in the experiment section, we can also learn that with a decrease in the thickness of the double-layer (*d*), the specific capacitance (*C*) will increase. Hence, the GSC(SC-40) sample is likely to form more double-layer capacitance. Moreover, SC can also lead to a shorter diffusion path for ion transportation in GSC(SC-40) and in the same way, improve the electrochemical performance.^22^ On the contrary, GSC(SC-100) and GSC(SC-600) have the opposite effect due to the remaining SC. In order to further confirm that GSC(SC-40) has excellent electrochemical reversibility and capacitance properties, the galvanostatic charge–discharge curves of GSC(SC-40) at different current densities of 0.1–5.0 A g^−1^ are shown in Fig. 5(d). All the curves are linear and symmetrical triangular shaped during the charge–discharge process. The specific capacitance of GSC and GSC(SC-40) is displayed in Table 1 at different current densities. The specific capacitance of GSC(SC-40) at all current densities is higher than that of GSC. Moreover, it is obvious that because of the same curled structure, the specific capacitance of GSC and GSC(SC-40) decreases with an increase in current density. Compared with GSC, GSC(SC-40) is completely curled and provides more electric double-layers, so the specific capacitance of GSC(SC-40) is higher than that of GSC at all current densities. We have also measured the cycling stability of GSC and GSC(SC-40) at a current density of 5 A g^−1^ after 3000 cycles as shown in Fig. S4.† After 3000 cycles, compared to GSC (88.9%), the GSC(SC-40) electrode exhibited excellent cycling stability and retained 94.6% of its initial specific capacitance. This suggests that GSC(SC-40) has excellent electric double-layer capacitor characteristics and can be used as a supercapacitor electrode material.

**Table 1:** Specific capacitance of GSC and GSC(SC-40) at different current densities

Current density (A g^−1^)	Specific capacitance (F g^−1^)	
	GSC	GSC(SC-40)
0.3 A g^−1^	172	245
0.5 A g^−1^	124	203
1.0 A g^−1^	107	178
5.0 A g^−1^	67	113

**Fig. 6:**
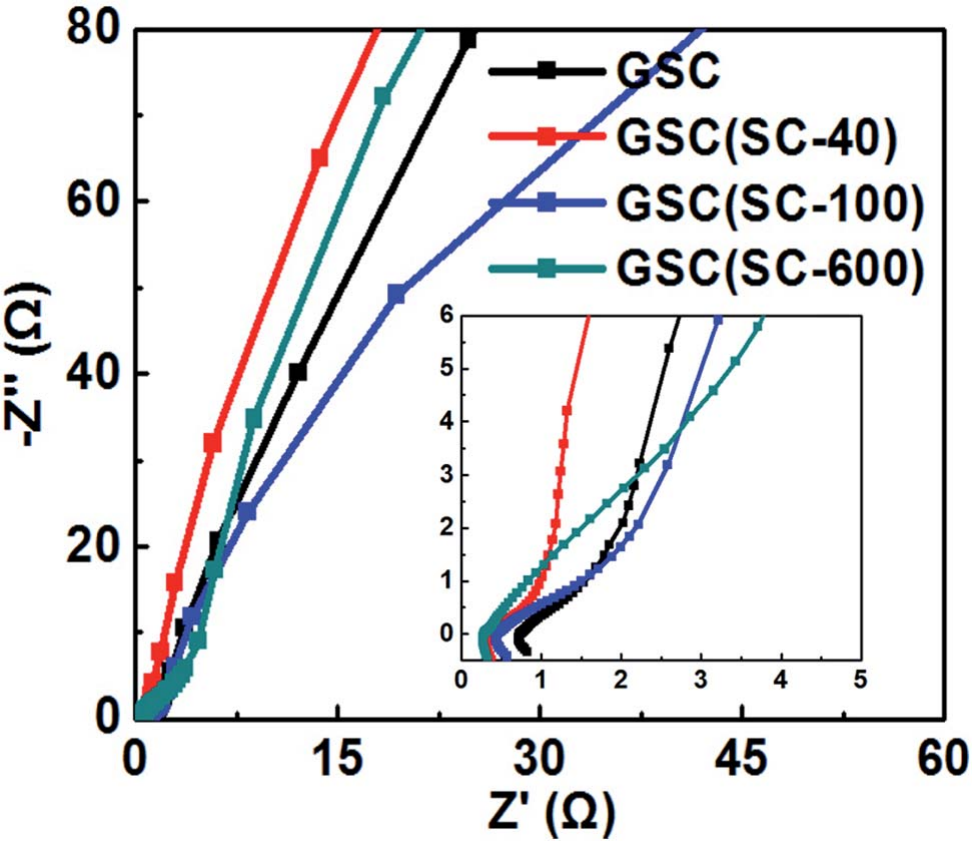
The Nyquist impedance plot of all the samples at the same open circuit potential.

The above results show that the addition of SC is an important factor for excellent electrochemical performance. The conductive performance of GSC(SC-40) may improve because of the appropriate scroll structure. So, the complex plane plots of the Nyquist impedance spectra for GSC, GSC(SC-40), GSC(SC-100) and GSC(SC-600) are shown in Fig. 6. The intercept at the real part (*Z*′) in the high-frequency range corresponds to the electrode series resistance of the ionic resistance of the electrolyte, the intrinsic resistance of the substrate and the contact resistance at the active material/current collector interface.^4^ In Fig. 6, there is an illustration showing the impedance plots in an expanded high-frequency range. It can be clearly seen that the intercept at the real part (*Z*′) for GSC(SC) is smaller than for GSC, indicating that GSC(SC) has good electrode conductivity and very low internal resistance.^4^ At high-medium frequency, the semicircle is associated with the faradaic charge transfer resistance (*R*_ct_).^26^ In the illustration of Fig. 6, the semicircle of GSC(SC-40) is the smallest of all the samples, showing that GSC(SC-40) has a fast charge transfer process. At lower frequencies, a straight sloping line represents the diffusive resistance (Warburg impendence) of the electrolyte in the electrode pores and proton diffusion in the host material.^26^ If the Nyquist plot is close to a vertical line, the active material will have nearly ideal capacitive behavior of the EDLC.^4^ For GSC(SC-40), the Nyquist plot is closer to a vertical line than the other samples, demonstrating that the GSC(SC-40) sample has nearly ideal capacitive behavior.

We have also tested the electrical conductivity of all the samples. The results show that the electrical conductivity of GSC(SC-40) (4.3 × 10^4^ S m^−1^) is higher than that of GSC (3.2 × 10^4^ S m^−1^). Conversely, when 100 and 600 mg SC is added, the electrical conductivity decreases and is 2.1 × 10^4^ S m^−1^ and 1.8 × 10^4^ S m^−1^, respectively. When too much SC is added, there are more agglomerations and remaining SC, which hinders electronic transmission and results in lower conductivity. The GSC(SC-100) and GSC(SC-600) samples show high resistance and poor electrochemical performance. These results are consistent with the above.

GSC(SC-40) with moderate SC has many excellent properties. The appropriate scrolled structure of GSC(SC-40) has characteristics such as open ends/edges and optimal interlayer distance. The porous structure and open ends/edges could provide convenient channels for the accumulation of ion charges at the electrode–electrolyte interface.^26^ The optimal interlayer distance should influence the electronic transport owing to the p–p interaction between the inner and outer surfaces of scrolled graphene, and could help to form double electric layer capacitors.^26^

## 4. Conclusions

In summary, we have successfully synthesized GSCs by a cold quenching method in liquid nitrogen. The number of layers and the curling degree for the GSCs could be controlled by adding SC to the GO aqueous suspensions during the preparation process. The quantity of SC added has a significant impact on the structure and the electrochemical properties of the GSCs. We found that adding a moderate amount of SC can enhance the interface between the GSs and lead to a shorter interlayer spacing, which forms more double layers. When the quantity of SC added is too much, a large amount of the SC is tightly wrapped in the GSCs and also remains after the thermal reduction process. For a supercapacitor, GSCs with moderate SC (GSC(SC-40)) shows better electrochemical properties than GSC, GSC(SC-100) and GSC(SC-600). So we can control the performance of the supercapacitor by controlling the structure of the GSCs.

## Conflicts of interest

There are no conflicts to declare.

## Supplementary Material

RA-008-C8RA02231C-s001
