# Supplementary material for: Controllable synthesis of graphene scrolls and their performance for supercapacitors
Source: RSC Adv. 2018 May 24;8(34):19164–70. doi: 10.1039/c8ra02231c (PMC9080597; doi:10.1039/c8ra02231c)
Supplement: RA-008-C8RA02231C-s001 [file RA-008-C8RA02231C-s001.pdf]

Electronic Supplementary Material (ESI) for RSC Advances.
This journal is © The Royal Society of Chemistry 2018

Supporting Information

Controllable Synthesis of Graphene scroll and their Performance for Supercapacitor

Lianlian Gao †, Zhigang Zhang †, Jinping Zhao, Jin Zhou, Zhichao Miao,Weijiang Si*, Shuping Zhuo*

† These authors contribute equally to this paper

* Corresponding author

School of Chemistry and Chemical Engineering, Shandong University of Technology, Zibo 255049, P. R. China

E-mail: jpzhao@sdut.edu.cn; siweijiang@sdut.edu.cn

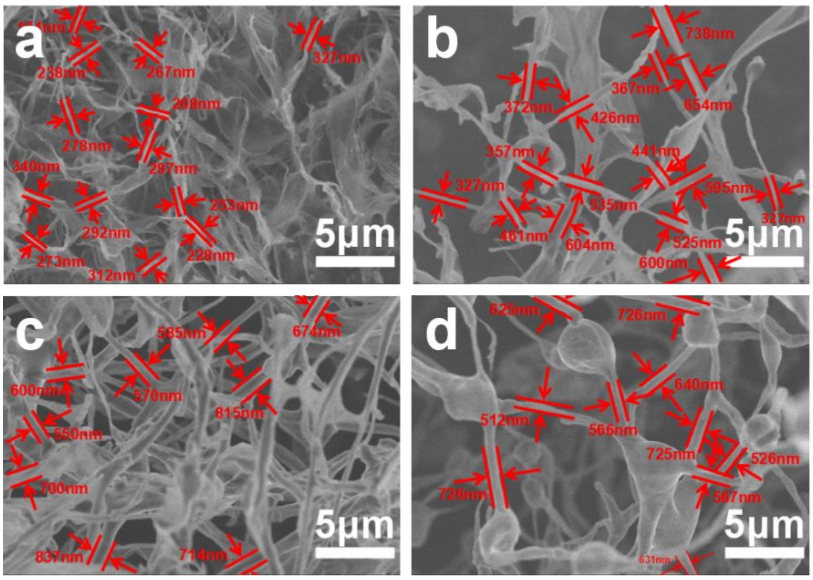

Fig. S1. SEM images of GSC (a), GSC(SC-40) (b), GSC(SC-100) (c) and GSC(SC-600) (d).

Tab. S1. The diameter distribution of GSC, GSC(SC-40), GSC(SC-100) and GSC(SC-600).

Diameter distribution (nm)	Sample			
	GSC	GSC(SC-40)	GSC(SC-100)	GSC(SC-600)
1	208	357	550	505
2	228	367	570	520

3	238	426	585	560
4	253	441	600	560
5	267	461	674	619
6	273	525	700	625
7	278	535	714	637
8	292	595	743	720
9	297	600	815	720
10	312	604	837	720
Average	265	491	679	619

Tab. S2. The element composition by XPS of GSC, GSC(SC-40), GSC(SC-100) and GSC(SC-600).

Sample	Element composition by XPS (at%)				
	C	O	C/O	Na	C/Na
GSC	90.67	9.33	9.72	---	---
GSC(SC-40)	89.64	9.27	9.67	1.09	82.24
GSC(SC-100)	69.56	21.87	3.18	8.57	8.12
GSC(SC-600)	71.74	25.44	2.82	9.36	7.66

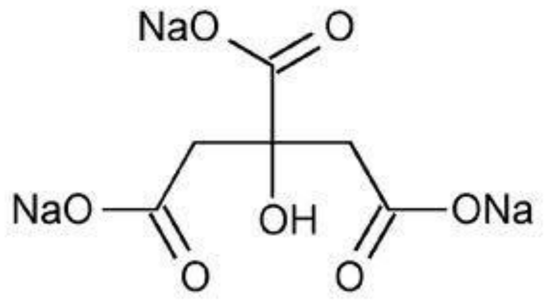

Fig. S2. The molecular formula of SC.

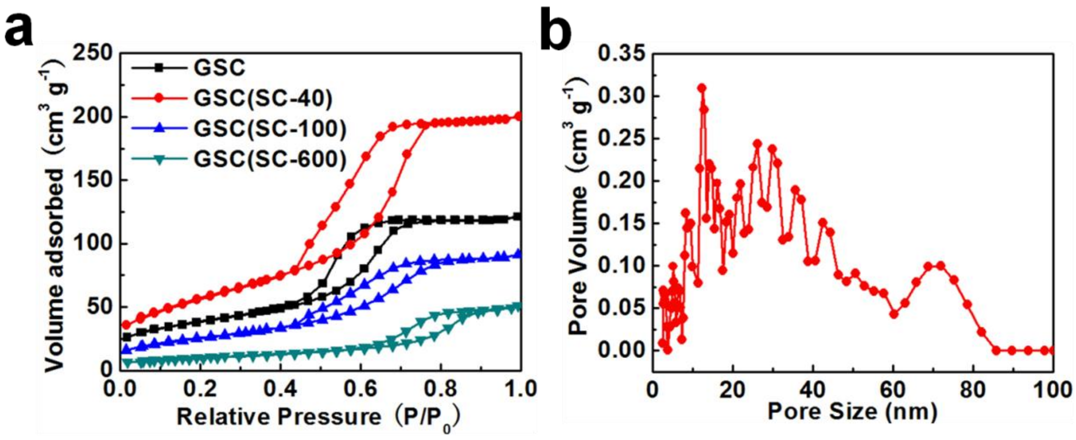

Fig. S3. (a) N_2 sorption isotherms; (b) pore size distribution plots of GSC(SC-40).

Tab. S3. S_{BET} of all prepared materials.

Sample	GSC	GSC(SC-40)	GSC(SC-100)	GSC(SC-600)
S_{BET}(m^2 g^{-1})	207	318	134	47

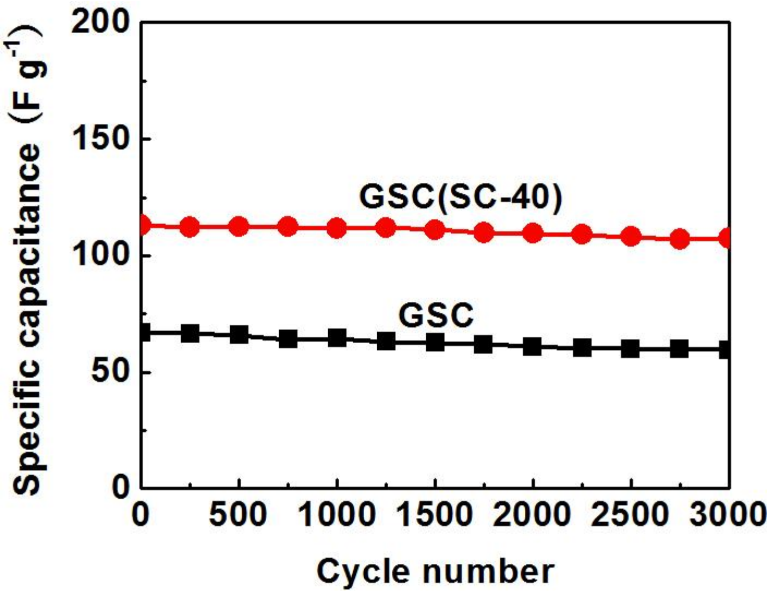

Fig. S4. Cyclic stability of GSC and GSC(SC-40) at current density of 5 A/g after 3000 cycles.
